# Mesenchymal Stromal Cells: Current Understanding and Clinical Status

**DOI:** 10.1002/stem.269

**Published:** 2009-12-04

**Authors:** Husein K Salem, Chris Thiemermann

**Affiliations:** Centre for Translational Medicine and Therapeutics, The William Harvey Research Institute, St. Bartholomew's and The Royal London School of Medicine and Dentistry, Queen Mary—University of LondonLondon, United Kingdom

**Keywords:** Stem cells, Mesenchymal stromal cells, T lymphocytes, Dentritic cells, Myocardium, Kidney

## Abstract

Multipotent mesenchymal stromal cells (MSCs) represent a rare heterogeneous subset of pluripotent stromal cells that can be isolated from many different adult tissues that exhibit the potential to give rise to cells of diverse lineages. Numerous studies have reported beneficial effects of MSCs in tissue repair and regeneration. After culture expansion and in vivo administration, MSCs home to and engraft to injured tissues and modulate the inflammatory response through synergistic downregulation of proinflammatory cytokines and upregulation of both prosurvival and antiinflammatory factors. In addition, MSCs possess remarkable immunosuppressive properties, suppressing T-cell, NK cell functions, and also modulating dentritic cell activities. Tremendous progress has been made in preclinical studies using MSCs, including the ability to use allogeneic cells, which has driven the application of MSCs toward the clinical setting. This review highlights our current understanding into the biology of MSCs with particular emphasis on the cardiovascular and renal applications, and provides a brief update on the clinical status of MSC-based therapy.

## INTRODUCTION

**Figure 1:**
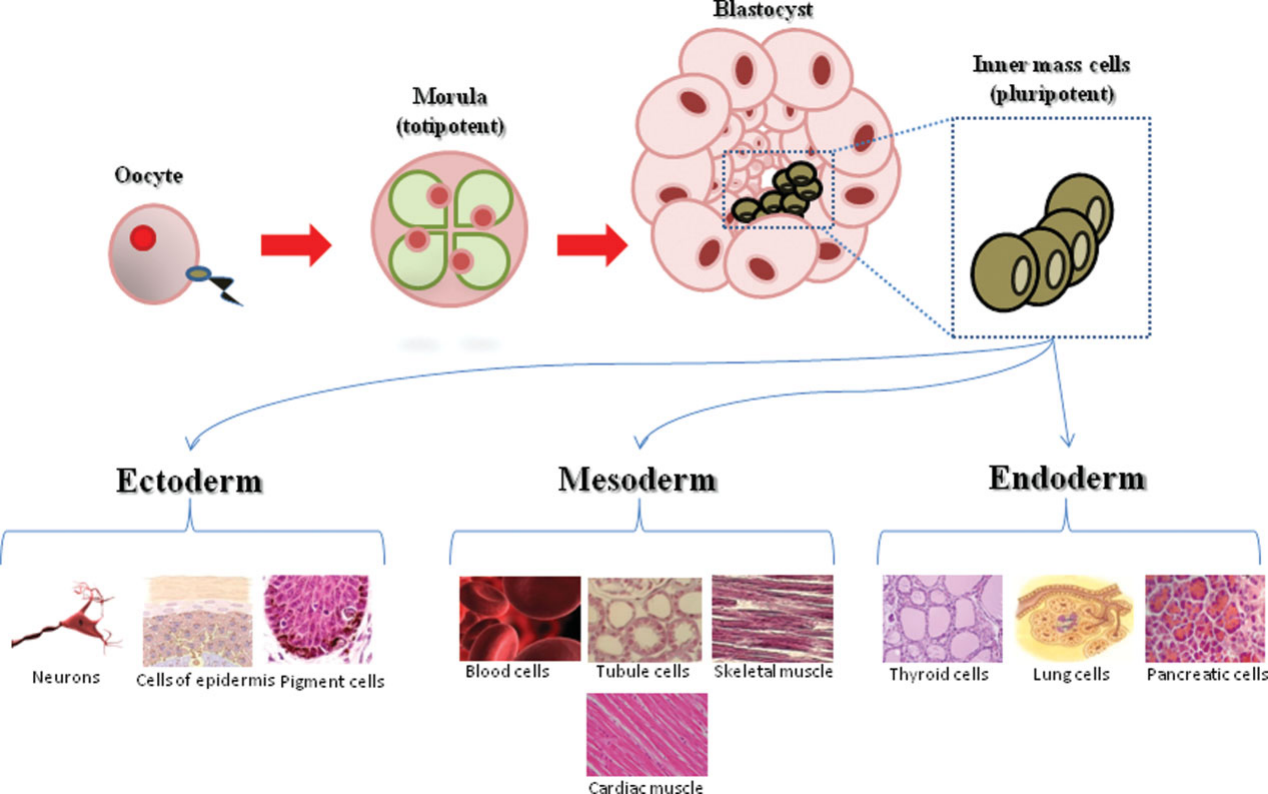
Hierarchy of stem cells.

The use of stem cells in the clinical arena has gathered tremendous momentum over the last decade, advanced by varying levels of success in clinical trials and by the advancement in our understanding of the mechanisms by which stem cells exert their seemingly favorable effects. Broadly speaking, stem cells can be characterized as either embryonic or adult stem cells. In theory, embryonic stem cells (ESs) appear to be the most versatile stem cell type for application in regenerative medicine. In the hierarchy of ESs, cells taken from the fertilized oocyte are called *totipotent* [1]. These totipotent cells are then able to specialize, forming the blastocyst from which the embryo will develop. ESs from within this blastocyst are called *pluripotent* as these cells go on to specialize to form all three of the germ layers (Fig. 1). Fully developed adult tissues and organs contain niches of multipotent adult stem cells. Originally these multipotent adult stem cells were described as being able to differentiate into varying cell lineages from within their respective germ layer [1]. The development of induced pluripotent stem cells (iPS) [2] and the characterization of adult stem cells differentiating into cell types of differing germ layers have complicated the nomenclature of adult stem cells and therefore, flexibility and caution is required when defining specific stem cell types. However, the key properties that stem cells exhibit are unlimited self-renewal and multilineage potential. The ethical issues surrounding the use of ESs, the lack of understanding about how to specifically regulate ES differentiation, and the widely reported tumorigenicity [3] associated with ESs in experimental models have, in part, driven researchers to develop and use adult stem cells that lack these side effects.

Adult stem cells have been described from a wide range of adult tissues, including the brain, heart, lungs, kidney, and spleen. However, the most well-characterized source for adult stem cells is still adult bone marrow. Adult bone marrow contains a heterogeneous population of cells, including hematopoietic stem cells, macrophages, erythrocytes, fibroblasts, adipocytes, and endothelial cells. In addition to these cell types, bone marrow also contains a subset of nonhematopoietic stem cells that posses a multilineage potential [4,5]. These stem cells are commonly called *marrow stromal stem cells* or *mesenchymal stem cells*, and more commonly now, *mesenchymal stromal cells* (MSCs). MSCs are primitive cells originating from the mesodermal germ layer and were classically described to give rise to connective tissues, skeletal muscle cells, and cells of the vascular system.

MSCs, in the traditional view, should refer to stem cells that are also capable of producing blood cells; however, blood cells are actually derived from a distinct cell population called the *hematopoietic stem cells*. This allows MSCs to be classified as *nonhematopoietic*, multipotential stem cells that are capable of differentiating into both mesenchymal and nonmesenchymal cell lineages.

More than 30 years ago, Friedenstein et al. [6] first reported evidence of fibroblast-like cells that could be isolated from bone marrow via their inherent adherence to plastic in culture. He described a population of cells as multipotential stromal precursor cells that were spindle-shaped and clonogenic in culture conditions, defining them as colony-forming unit fibroblasts (CFU-F). These cells were able to differentiate into adipocytes, chondrocytes, osteocytes, and myoblasts, both in vitro and in vivo. In addition, it has also been demonstrated that MSCs are capable of differentiating into cardiomyocytes, neurons, and astrocytes in vitro and in vivo [5,7–10]. These observations have formed the basis for most of the current studies of bone marrow-derived stromal cells. However, there still remain many unanswered questions about the true nature and identity of MSCs, including location, origin, and multipotential capacity. Isolation of MSCs has been reported from several tissues, including adipose tissue, liver, muscle, amniotic fluid, placenta, umbilical cord blood, and dental pulp [5,10–12], however bone marrow remains the principal source of MSCs for most preclinical and clinical studies.

The true identity of MSCs has often been confused by different laboratories which employ different isolation and in vitro culture methods. These variables are responsible for the phenotype and function of resulting cell populations. Whether these conditions selectively promote the expansion of different populations of MSCs or cause similar cell populations to acquire different phenotypes is not clear. It is estimated that MSCs represent only between approximately 0.01 and 0.001% of the total nucleated cells within isolated bone marrow aspirates [8,13]. Despite this low number, there remains a great interest in these cells, as they can easily be isolated from a small aspirate and culture-expanded through as many as 40 population doublings to significant numbers in about 8 to 10 weeks. MSCs have been studied from different sources, and each type has been reported to vary in their proliferative and multilineage potential. In addition, the lack of any single unique specific cell surface marker to identify this cell population, coupled with differences in terminology, has hindered the progress of MSC research. Position papers from the International Society for Cellular Therapy have attempted to address these issues by clarifying the terminology and calling the cells multipotent MSCs that should include the source in the terminology, that is, adipose-derived MSCs, bone marrow-derived MSCs, etc. The International Society for Cellular Therapy has also provided the following minimum criteria for defining multipotent human mesenchymal stromal cells [14]:

1. plastic-adherent under standard culture conditions;
2. positive for expression of CD105, CD73, and CD90, and absent for expression of hematopoietic cell surface markers CD34, CD45, CD11a, CD19, and HLA-DR;
3. under specific stimulus, cells should differentiate into osteocytes, adipocytes, and chondrocytes in vitro.

Table 1 lists many cells' surface markers that have been used to characterize MSCs as either positive for or negative for expression [8,15,16], although this is further complicated by the differences between species and between different strains of species [17]. This issue remains unresolved in the absence of the identification of a unique cell surface marker. The current evidence supporting the use of MSCs as a biologic therapeutic for a diverse range of clinical applications includes: ease of accessibility for isolation, enormous expansion potential in culture, presumptive plasticity, immunosuppressive properties, use in allogeneic transplantation, paracrine-mediated effects, homing and migratory behavior to sites of tissue injury, and ethical considerations. This review aims to highlight the current state of our knowledge, in particular the cardiovascular and renal preclinical studies and the status of clinical trials involving MSC therapies.

**Table 1:** Markers for the isolation of mesenchymal stromal cells

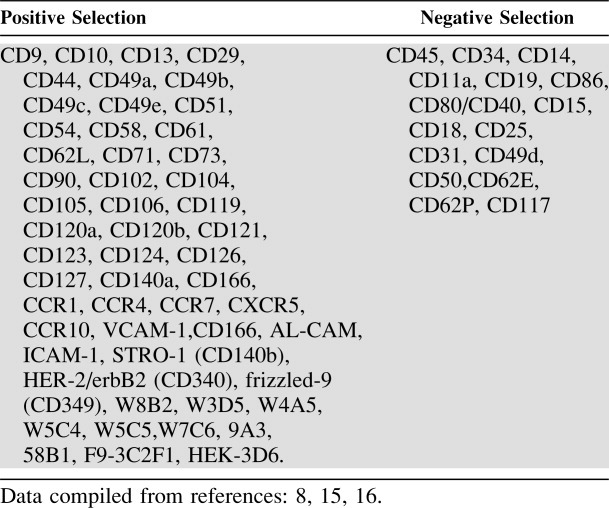

### Localization and Trafficking of MSCs

MSCs reside in specialized niches within various tissues, and it has been shown that bone marrow, bone, and spleen are all sites of engraftment [18–21]. It has also recently been reported that a very small number of MSCs consistently circulate in the peripheral blood under stationary conditions, and that this circulating pool is greatly increased under hypoxic conditions [20]. However, several studies have reported contrasting results and the inability to locate any circulating MSCs at all (reviewed in [22]).

The use of MSCs for therapeutic applications has been particularly hailed because of their presumed inherent ability to home to sites of inflammation following tissue injury when injected intravenously. Chapel et al. [23] demonstrated in a model of multiple organ failure that green fluorescent protein (GFP)-tagged MSCs homed to numerous tissues with localization correlating to the severity and geometry of injury. Homing is essentially the process by which cells migrate to and engraft in the tissue in which they will exert functional and protective effects. This homing feature of MSCs means that the presumed complications associated with intramuscular or site-specific injection of stem cells, such as ossifications [24], is avoided, and systemic intravenous delivery with the potential for multiple dosages is possible.

Although the precise molecular mechanisms by which MSCs are able to migrate and home into sites of injury are not yet fully understood, the complex multistep process by which leukocytes migrate to peripheral sites of inflammation has been proposed as a paradigm (leukocyte adhesion cascade reviewed [25]). This serves as a useful, albeit somewhat simplified comparison, especially since more recent studies have proposed a plethora of additional pathways and processes involved in the migration, trafficking, and engraftment of MSCs.

**Figure 2:**
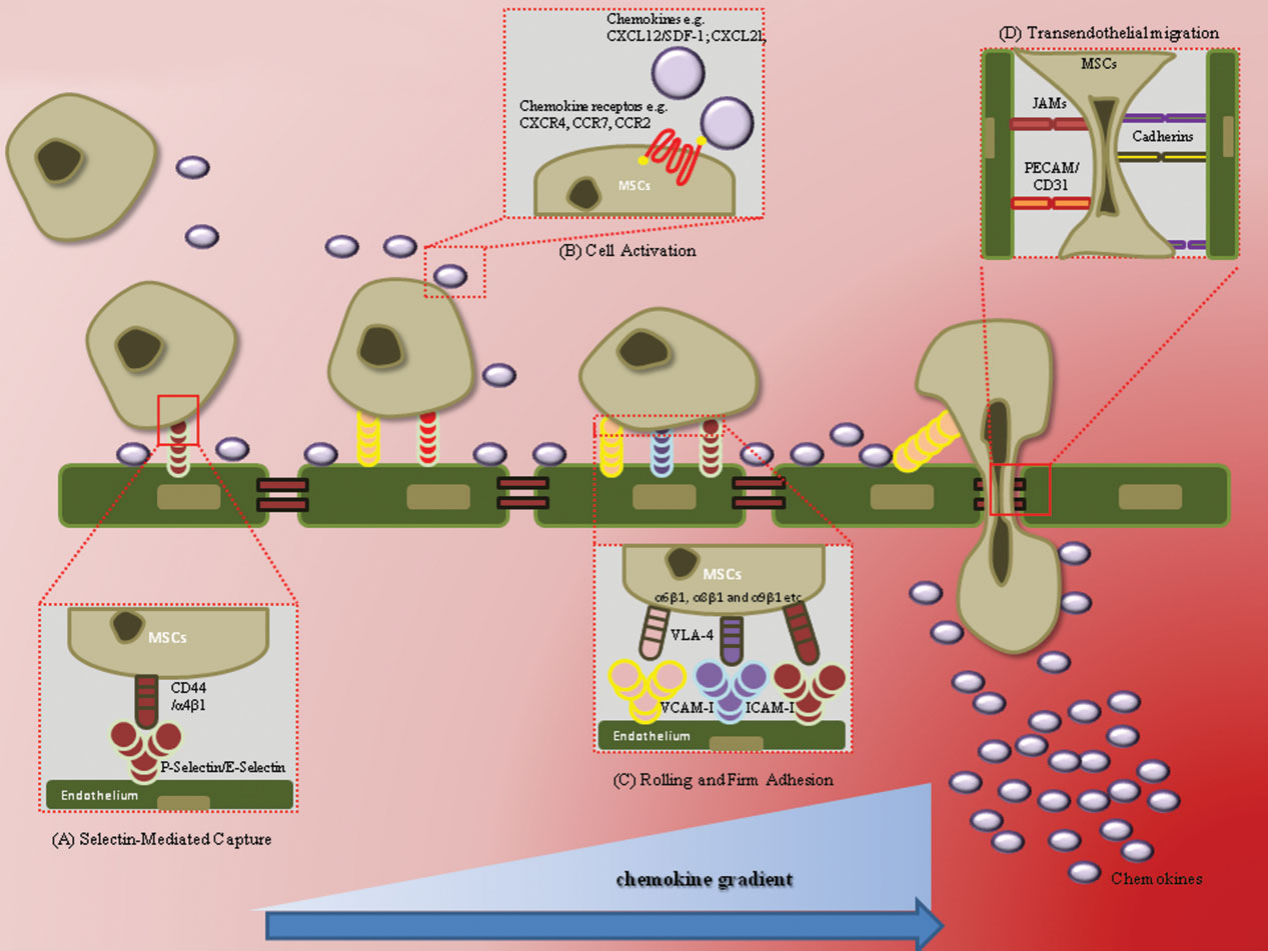
Proposed mechanisms involved in the homing and trafficking of mesenchymal stromal cells to sites of tissue injury after infusion. Abbreviations: ICAM, intercellular adhesion molecule; JAMs, junctional adhesion molecules; MSCs, mesenchymal stromal cells; PECAM, platelet-endothelial cell-adhesion molecule; PGE, prostaglandin E_2_; VCAM, vascular cell-adhesion molecule; VLA, very late antigen.

During inflammation, the recruitment of inflammatory cells requires a coordinated sequence of multistep adhesive and signaling events, including selectin-mediated rolling, cell activation by chemokines and cytokines, activation of integrins, integrin-mediated firm adhesion on endothelium, transendothelial migration, and finally the migration/invasion in the extracellular matrix involving integrin-dependent interactions and matrix-degrading proteases [26,27] (Fig. 2). It is well known that migratory direction follows a chemokine density gradient. The increase in inflammatory chemokine concentration at the site of inflammation is a key mediator of trafficking of MSC to the site of injury. Chemokines are released after tissue damage and MSC express several receptors for chemokines [28]. Activation by such chemokines is also an important step during trafficking of MSCs to the site of injury. SDF-1/CXCL12 is a member of the chemokine family and is constitutively expressed by bone marrow stromal cells and other progenitor cells [29]. Early passage MSCs have been shown to express the specific SDF-1 chemokine receptor CXCR4 [30,31]. In addition, MSCs also express several adhesion molecules [8,32,33] which respond to SDF-1 as well as chemokines CX3CL1, CXCL16, CCL3, CCL19, and CCL21 [21,34]. SDF-1 has been shown to stimulate not only hematopoietic stem cell engraftment, but also the recruitment of other progenitor cells, including MSCs, to the site of tissue injury [35]. Inhibition of the SDF-1/CXCR4 axis partially blocks the homing of CXCR4-expressing cells to the site of injury [36,37]. Endothelial nitric oxide synthase (eNOS)-derived nitric oxide (NO) production from the host myocardium has recently been described to promote MSC migration toward the ischemic myocardium via upregulation of SDF-1, with MSC trafficking toward the region of ischemia leading to improved cardiac function [38]. Furthermore, overexpression of SDF-1 was demonstrated to enhance stem cell homing and incorporation into ischemic tissues [39], suggesting that SDF-1 plays a crucial role for recruitment of intravenously-infused cells.

Although the SDF-1/CXCR4 axis has been well characterized as a pathway for MSC homing, several other ligand-receptor interactions have also been recently reported to be involved in MSC homing. Sasaki et al. [40] recently demonstrated that injected-MSCs significantly contribute to wound repair via MSC accumulation in the wound site. Keratinocytes within sites of wounded skin have been reported to express CCL21 [41], and MSCs, characterized to express the CCL21-specific receptor CCR7, were reported to recruit to the wound site via the specific CCL21/CCR7 interaction, both in vitro and in vivo [40]. Furthermore, it was shown that local intradermal delivery of CCL21 may have also contributed to the differentiation of MSCs to multiple cell types [40].

Integrins have also been reported to play a key role in cell adhesion, migration, and chemotaxis [26]. Ip et al. [42] identified integrin β1 as a distinct pathway and not CXCR4, in a model of AMI, for trafficking and engraftment of MSCs to the ischemic myocardium. An alternate distinct pathway identified involving specific integrin-mediated trafficking has also recently been reported. Podocalyxin (PODXL) is a member of the CD34 family of membrane mucin-proteins [43]. Lee et al. [44] recently demonstrated the role of PODXL and integrin α6 (CD49f) in MSC migration and homing; and demonstrated MSCs engrafted more efficiently in both the injured heart and kidney. Using FACS analysis, it was reported that culture-expanded PODXL(hi)/CD49f(hi) MSCs were more clonogenic and differentiated more efficiently than PODXL(low)/CD49f(low) cells. Inhibition of expression of PODXL with RNA interference caused aggregation of the cells [44]. Furthermore, PODXL(hi)/CD49f(hi) MSCs were less prone to produce lethal pulmonary emboli, and significantly greater numbers of cells were recovered in heart and kidney following intravenous infusion into mice with myocardial infarcts [44].

The homing efficiency of MSCs has been reported to be greatly influenced by the variation in protocols currently used to isolate and culture expand populations to significant numbers required for in vivo use. It has been suggested that subculturing of MSCs may potentially lead to changes in their phenotype that effects MSC homing [45] and progressive subculturing has been associated with a decrease in expression of adhesion molecules, the loss of chemokine receptors, including CXCR4, and a subsequent lack of chemotactic response [30,46].

Many groups have attempted to modify the functional properties of MSCs to increase their homing potential. Modification of MSCs has included transduction of MSCs with CXCR4 using a retroviral vector [37] and treatment of the cells with tumor necrosis factor-α (TNFα), interferon-β and –γ (IFN-β –γ), copaxone [47], and insulin-like growth factor [48]. In addition, some groups have reported the use of alternative culturing protocols to modify gene expression of chemokine receptors such as CXCR4. It was reported that transfer of a cultured monolayer of MSCs to a “hanging drop” method resulted in spontaneous association of cells leading to formation of spheroids. This led to substantial changes in the gene expression pattern, including upregulation of CXCR4 and the α2 integrin subunit mRNAs [49].

The initial homing events involve the processes of rolling and tethering upon the endothelium between E- and P-selectin (considered as critical molecules for the rolling process) [50]. These are constitutively expressed by bone marrow endothelial cells and on endothelium in inflamed tissue [51]. Physiologic selectin receptors constitutively express sialylated residues such as PSGL-1 and CD44 [52]. CD44 is known to be highly expressed by MSCs. Rolling is subsequently followed by arrest and firm adhesion, with chemokines receptors expressed on the surface of endothelium ligating to respective chemokines and activating integrins, such as very late antigen-4 (VLA-4) (also known as α4β1-integrin) [53], which in turn bind to their ligands mediating firm adhesion. Ruster et al. [54] also reported that MSCs bind to endothelial cells in a P-selectin dependent manner and that rolling MSCs engage VLA-4 and vascular cell-adhesion molecule one (VCAM-1) to mediate firm adhesion to the endothelium. Firm adhesion is followed by transendothelial migration between endothelial cells via the action of junctional adhesion molecules (JAMs), cadherins, and platelet-endothelial cell adhesion molecule-1 (PECAM-1/CD31), mediating translocation to the extracellular matrix where they adhere to the extracellular matrix through molecules such as collagen, fibronectin via α1 integrins, hyaluronic acid, and CD44 (Fig. 2).

The inhibition of both MMP (matrix metalloproteinase) and TIMP-1/2 (tissue inhibitor of metalloproteinase) through gene knockout studies have been shown to attenuate MSCs migration though the basement membranes, and it was reported that these proteins are triggered by inflammatory cytokines [55,56]. Steingen et al. [57] also demonstrated the role of VCAM-1 and VLA4 to be involved in the extravasation process. In addition, MSCs have been shown to express combinations of integrins α6β1, α8β1, and α9β1 which are likely contributors to this process [42]. A clearer understanding of the exact mechanisms by which MSCs home to sites of tissue inflammation is likely to identify further opportunities to improve the clinical benefits observed after transplantation via their homing/migratory capacity.

Although numerous preclinical and clinical studies have demonstrated the safe and toxicity-free effects of MSC transplantation, many reports suggest that there exists a clear similarity between stem cell and cancer stem cell genetic programs [58]. It has been reported that over longer term ex vivo culture-expansion periods, human MSCs can undergo spontaneous transformation [59]. Miura et al. [60] reported that long-term cultured MSCs that had undergone spontaneous transformation colonized to multiple organs when delivered intravenously in vivo. These studies have important implications in assessing the safety profile of MSC-based therapies and the clear requirement for long-term follow-up studies on clinical trials that are currently ongoing.

### Immunomodulatory Capacity of Mesenchymal Stromal Cells

MSCs have received renewed interest, particularly in their use of transplantation medicine. Although traditionally the regenerative capacity of MSCs through their presumptive plasticity was seen as the driving force behind interest in MSCs, their role in modulating the immune response is now attracting greater interest. T lymphocytes (T-cells) are a major executor of the adaptive immune response, and numerous studies have demonstrated that MSCs modulate the function of T-cells. MSCs lack expression of MHC class II and most of the classical costimulatory molecules such as CD80, CD86, or CD40 [4,8,61]. MSCs can, however, express class II molecules under specific conditions [33]. Le Blanc et al. [62] showed that MHC class II antigens present inside MSCs can be expressed on the cell surface after induction with interferon gamma (IFN-γ). This is relevant because, in many inflammatory milieus, IFN-γ is upregulated, which in turn may result in an increase in the expression of MHC class II. However, pretreatment of MSCs with IFN-γ failed to generate a proliferative response in allogeneic lymphocytes [63], and MSCs expressing MHC class II antigens also failed to elicit a proliferative response [61]. In addition, the lack of expression of T-cell costimulatory molecules suggests that T-cell activity may result in anergy (immune unresponsiveness) that may contribute to the observed immune tolerance [33]. In contrast, however, Stagg et al. [64] reported that IFN-γ stimulated syngeneic MSCs acted as conditional antigen-presenting cells (APCs) which were able to activate antigen-specific immune responses, suggesting important implications in the development and selection of either autologous or allogeneic MSC therapeutics.

The traditional view that MSCs simply evaded the host immune response is not quite as simple as first proposed. MSCs have been characterized as expressing several receptors that allow them to interact with T-cells. MHC class I and several adhesion molecules, including cell adhesion molecule (VCAM), intercellular adhesion molecule one (ICAM-1), activated leukocyte cell adhesion molecule (ALCAM), lymphocyte functional antigen-3 (LFA3), and some integrins can interact with their respective ligands on T-cells [65]. MSCs have also been reported to express functionally active indoleamine 2,3-dioxygenase (IDO) following stimulation by IFN-γ. IDO catalyzes the conversion from tryptophan to kynurenine [66], and this has been identified as a T-cell inhibitory effector pathway [67,68].

Miesel et al. [69] demonstrated that MSCs that expressed functional IDO protein were able to inhibit allogeneic T-cell responses in mixed lymphocyte reactions. IDO activity resulted in tryptophan depletion and kynurenine production as detected in coculture supernatants, and furthermore, the inhibitory action could be reversed by the addition of tryptophan.

Production of nitric oxide (NO) by MSCs has also been implicated as a potential mechanism by which MSCs inhibit T-cell proliferation [70]. MSCs also appear to reduce T-cell activation through indirect mechanisms by inhibiting the maturation of dentritic cells (DCs) from monocytes. DCs have a fundamental role in antigen presentation to naive T-cells immediately after maturation, which can be induced by inflammatory cytokines. MSCs inhibit the maturation of monocytes (in addition to cord blood and CD34+ hematopoietic stem cells) into DCs [71,72].

Zhang et al. [73] reported that MSCs inhibited the upregulation of CD1a, CD40, CD80, and CD86 during DC maturation. MSCs also reduce the proinflammatory potential of DCs by inhibiting their secretion of TNF-α, IFN-γ, and interleukin (IL)-12, and conversely increasing levels of IL-10, inducing a more anti-inflammatory DC phenotype [71,74,75].

The interaction between MSCs and natural killer (NK) cells may contribute to the immunomodulatory effects of MSCs. NK cells are key effector molecules of innate immunity. MSC may evade recognition by alloreactive cytotoxic T-cells (CTL) and NK cells as these were not lysed in coculture experiments [76]. Angoulvant et al. [77] suggested this was mediated by the secretion of soluble factors by MSCs and through inhibition of CTL differentiation from precursors. However, NK cells can effectively lyse MSCs, despite the high levels of expression of MHC class I on MSCs [78]. Sotiropolou et al. [79] suggested a combinatorial effect on the suppressed proliferation of NK cells via cell-cell contact between MSCs-NK cells and secretion of soluble factors by MSCs including transforming growth factor beta (TGF-β) and prostaglandin E_2_ (PGE_2_).

**Figure 3:**
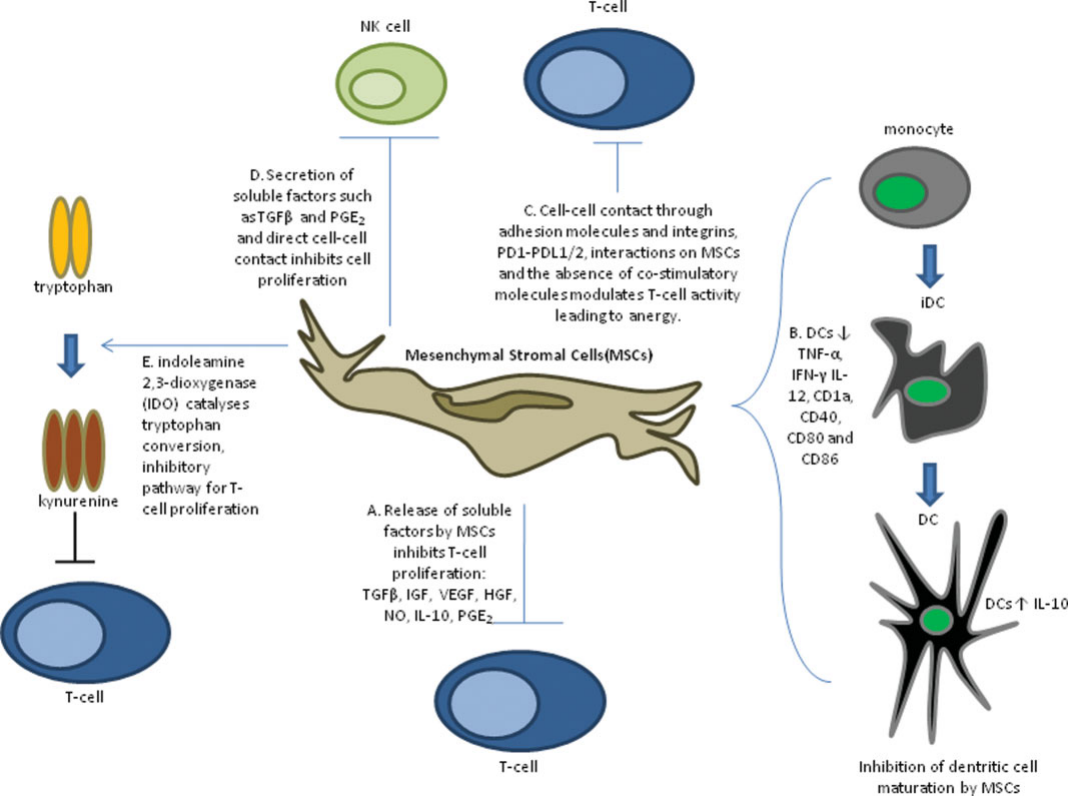
Immunomodulation by mesenchymal stromal cells. Abbreviations: DCs, dentritic cells; HGF, hepatocyte growth factor; ICAM, intercellular adhesion molecule; IGF, insulin-like growth factor; IL, interleukin; JAMs, junctional adhesion molecules; MSCs, mesenchymal stromal cells; NK, natural killer; NO, nitric oxide; PD-1, programmed death one; PD-L1, programmed death one ligand; PGE_2_ prostaglandin E_2_; T-cell, T lymphocyte; TGFβ, transforming growth factor beta; TNFα, tumor necrosis factor-α; VEGF, vascular endothelial growth factor; VLA, very late antigen.

The inhibitory molecule programmed death one (PD-1) binding to its ligands PD-L1 and PD-L2 may also be responsible for inhibition of T-cell proliferation via cell-cell contact of MSCs, leading both to modulate the expression of cytokine receptors and activate molecules for cytokine signaling [80,81]. The mechanisms by which MSCs exert their function on immune cells are pleiotropic and redundant, and it is clear that our understanding is far from complete. Figure 3 summarizes some of the multitude of possible effects of MSCs on immune cells.

### Use of MSCs in Renal Disease

Many kidney disorders involve both ischemic/inflammatory and immunologic injury. Therefore cell-based therapies such as those using MSCs which function through multiple mechanisms and have the potential to target the inflammatory and immunologic pathways have been considered a clinically relevant solution in contrast to pharmacologic agents that target only a single event or pathway in the pathophysiology of a given disease. The significant morbidity from long-term palliative treatments (that is, dialysis) and the ever increasing transplant waiting lists dictate a need for alternative therapy options such as cell therapy.

Chronic kidney disease results in significant cell loss, accumulation of extracellular matrix proteins, and development of interstitial fibrosis [82]. In contrast to conventional therapies that aim to limit these processes, cell therapy is focused more on the mechanisms promoting cellular repair and tissue remodeling. Many early studies reported that injury promoted the incorporation of bone marrow-derived cells into organs [83]. These observations supported the rationale that the incorporation of bone marrow-derived cells is part of and may aid the organ repair process. Ito et al. [84] reported that MSCs homed to injured kidneys and facilitated repair when chimeric rats carrying green fluorescent protein (GFP)-positive bone marrow cells were treated with anti-Thy1 antibody to induce glomerulonephritis and the mesangium was repopulated with GFP+ cells, mainly of nonhematopoietic lineages. Ischemia-reperfusion injury is one of the major causes of acute kidney injury, resulting in functional and structural changes in the kidney, mainly at the endothelium and proximal tubule cells [85–87].

Morigi et al. [88] reported that in an acute renal failure (ARF) model, injection of MSCs of male bone marrow origin protected cisplatin-treated syngeneic female mice from renal function impairment and severe tubular injury. Donor cells were shown to have localized in the context of the tubular epithelial lining and displayed binding sites for Lens culinaris lectin, suggesting that MSCs engrafted within the damaged kidney and differentiated into tubular epithelial cells, supporting renal structure and function recovery [88]. Lange et al. [89] reported that in an ARM model, MSC-treated animals had both significantly better renal function on days 2 and 3 and better injury scores at day 3 after ARF. Infusion of MSCs enhanced recovery of renal function and showed cells were located in the kidney cortex after injection. Togel et al. [90] reported that intracarotid administration of MSCs after renal ischemia resulted in significantly improved renal function, higher proliferative and lower apoptotic indexes, lower renal injury, and unchanged leukocyte infiltration scores compared with animals treated with syngeneic fibroblasts. These findings suggested that the renoprotective effects observed were mediated via a paracrine effect caused by the significant reduction in expression of proinflammatory cytokines IL-1β, TNF-α, IFN-γ, and iNOS, and the significant upregulation of anti-inflammatory cytokines IL-10, bFGF, TGF-α, and Bcl-2 in treated kidneys [90].

MSCs from GFP^+^ transgenic mice injected intravenously were reported to home to the kidney of mice with glycerol-induced ARF, but not in normal mice. These cells were shown to localize in the context of the tubular epithelial lining and expressed cytokeratin, suggesting that MSCs engrafted in the damaged kidney were able to differentiate into tubular epithelial cells. In addition, it was reported that MSCs enhanced tubular proliferation as detected by the increased number of proliferating cell nuclear antigen (PCNA) positive cells with a significant contribution of the engrafted MSCs in the regeneration of tubular epithelial cells demonstrated by the presence of a consistent number of GFP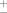 tubular cells 21 days after the induction of injury [91]. Crop et al. [92] recently demonstrated that donor-derived MSCs were capable of inhibiting recipient alloactivated T-cell proliferation before and after kidney transplantation. It was suggested that these immunosuppressive effects by MSCs were mediated by both cell-cell contact and release of soluble factors such as IL-10 and IDO.

These studies highlight the controversy that still remains about the exact mechanisms by which MSCs exert these seemingly beneficial effects. Held et al. [93] reported that in a mouse model of chronic renal injury, up to 50% of the regenerated proximal tubular epithelium resulted via cell fusion and not transdifferentiation following BM-derived cell transplantation. In addition, it was demonstrated that host epithelial cells underwent genetic modifications leading to repopulation of damaged epithelium and correction of renal disease. Both mechanisms of repopulation (bone marrow-derived renal tubule epithelium or by genetic alteration of endogenous cells) illustrate selection of functional cells leading to disease correction in the proximal tubules of the kidney [93]. While earlier studies suggested that MSCs engraft in the damaged kidney and differentiate into tubular epithelial cells resulting in restoration of renal structure and function [88,91], studies using an ischemic reperfusion (I/R) model of ARF provide evidence that MSCs did not differentiate into tubules, with labeled MSCs detected mostly in glomeruli and not in tubules or vascular endothelial cells [89,90]. These studies describe that MSC treatment was associated with improvement of renal function occurring within the first few days, suggesting a process independent of transdifferentiation into functional renal cells, and were more probably attributed to paracrine actions of these MSCs. This is supported by a study using a rat model of glomerulonephritis that suggested paracrine growth factor release rather than cellular differentiation was responsible for accelerated glomerular recovery in MSC-treated animals [94].

### Use of MSCs in Cardiovascular Disease

Cardiovascular disease (CVD) remains a significant cause of morbidity and mortality in the developed world. Coronary heart disease (CHD) is also the primary cause of clinical heart failure, and despite advances in medical therapy, 40% of patients with heart failure die within the first year of diagnosis [95]. Although pharmacologic, percutaneous coronary interventions and surgical interventions (for example, coronary artery bypass graft surgery) for CVD have improved outcomes, the rate of incidence continues to increase (http://www.who.int). In addition, cardiac transplantation is complicated by immunosuppressive therapy and is restricted by the limited supply of donor hearts. As with other degenerative diseases, the potential of cell therapy using stem cells to differentiate into viable cardiac myocytes and regenerate scar tissue is an attractive prospect, with the aim of reversing ventricular remodeling, preventing heart failure, and alleviating the need for heart transplantation.

Acute myocardial infarction (AMI) is associated with the death of cardiomyocytes by apoptosis and necrosis [96–98]. Although it has been reported that the heart exhibits some regenerative potential [99–101], it lacks the capacity to replace the significant cardiomyocyte losses caused by AMI that is subsequently compensated by cardiomyocytes hypertrophy and fibrosis. This remodeling process is associated with reduced ventricular compliance, ventricular dilatation, and eventually heart failure [102]. Clinical end-stage heart failure requires ventricular assist device implantation or, ideally, cardiac transplantation. With limited donor supplies and long-term patency issues associated with assist devices, cell therapy using MSCs to transdifferentiate into viable cardiomyocytes and regenerate scar tissue is an attractive prospect. Several groups have reported that MSCs, once exposed to a variety of physiologic or nonphysiologic stimuli, are capable of differentiating into cells displaying several features of cardiomyocytes-like cells [103–107]. In preclinical studies using experimental models of cardiac injury, MSCs can engraft after systemic administration and improve the repair of infarcted myocardium in rodents. In a porcine myocardial infarction (MI) model, bone-marrow derived MSCs (injected directly into the myocardium) efficiently engrafted into the host myocardium and showed evidence of myogenic differentiation within 2 weeks, which correlated with a significant reduction in infarct size, wall thinning, and contractile dysfunction [108].

In a rat model of dilated cardiomyopathy, transplantation of MSCs led to a significant increase in capillary density (enhanced angiogenesis) and a significant inhibition of myocardial fibrosis. Both myogenic and angiogenic differentiation of MSCs were observed, in addition to characterizing the secretion of several prosurvival growth factors, including vascular endothelial growth factor, hepatocyte growth factor, and insulin-like growth factor, suggesting that the benefits observed were caused by a combination of differentiation and paracrine mediated effects [109]. Jiang et al. [110] reported that the direct injection of MSC into the infarct border zone in a rat model improved cardiac function and caused a significant reduction in myocyte apoptosis as well as an increase in vessel density. Cardiac improvement was reported to be marked after transplantation at 1 week after MI compared with 1 hour or 2 weeks after MI, indicating that timing of cell delivery is equally critical for successful therapy [110]. The appropriate delivery method for MSC treatment is still very controversial. Several delivery approaches have been reported, including intravenous infusion [111], direct injection in the ventricular wall [112], transendocardial injection [113,114], and transcoronary artery injection [115] (Table 2).

**Table 2:** Clinical trials using mesenchymal stromal cells

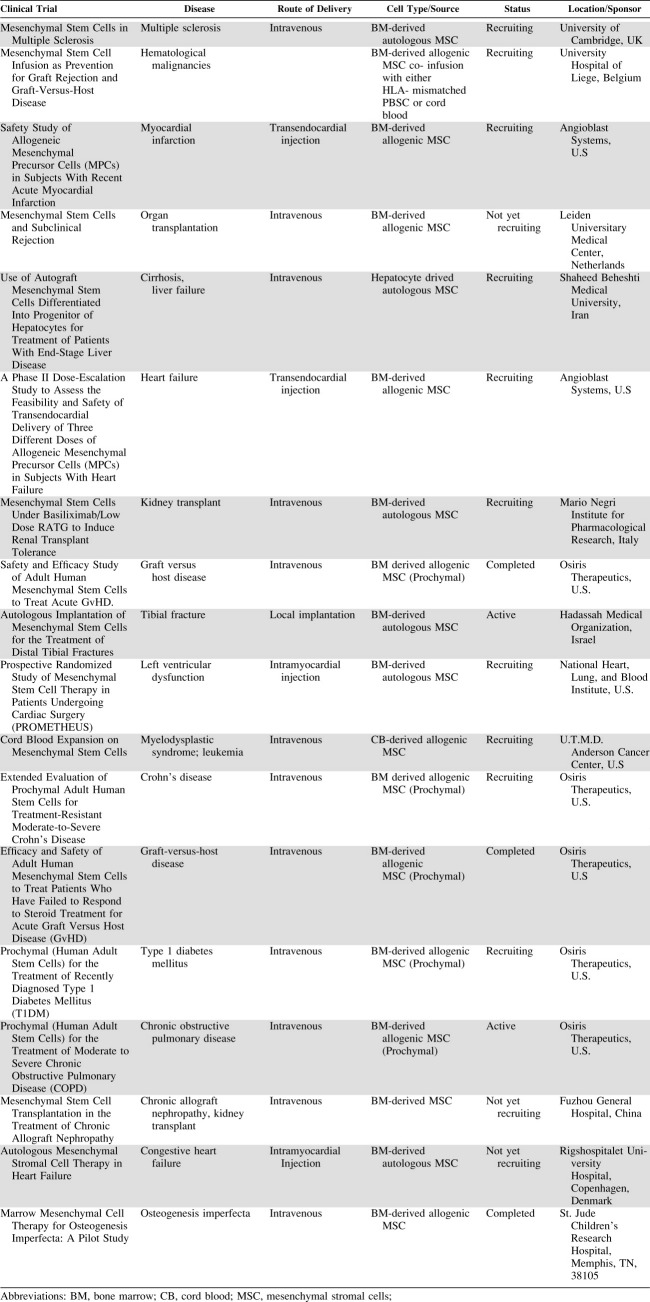

The question of the ideal route of administration remains one of the many unresolved issues facing efficient clinical application of MSCs. Clinical trials in humans aimed at improving cardiac function using stem cells have demonstrated variable but encouraging results [116]. Intracoronary injection of autologous MSCs was demonstrated to enhance left ventricular functional recovery after myocardial infarction [117–119]. Improvement of myocardial contractility was also demonstrated in approximately 50% of patients with MI after transcoronary transplantation of autologus MSC and endothelial progenitor cells in the infarcted area through the left anterior descending artery [115].

Table 2 summarizes the current clinical status of trials using MSCs. While initial studies demonstrate some beneficial effects, the mechanisms responsible for these effects are unclear, although differentiation of transplanted MSC into cardiomyocytes or their fusion with the host cardiomyocytes in vivo has recently been excluded [120,121]. Conversely, it has been suggested that the transplanted MSCs enhance angiogenesis in the ischemic tissues by secreting paracrine factors, including angiogenic cytokines and antiapoptotic factors [122,123].

### Clinical Application of MSC-Based Therapy

The therapeutic potential of MSCs is currently being explored in a number of phase I/II and III clinical trials, many of which have recently been completed or are underway (Table 2). Several of these studies investigated the use of MSC therapy to mediate HSC engraftment and reduce or eliminate graft-versus-host disease (GvHD). Recently it was demonstrated that infusion of culture-expanded haploidentical MSCs into unrelated pediatric umbilical cord blood transplantation recipients could be performed safely, and no adverse effects or associated toxicity were reported. The study demonstrated that all patients achieved neutrophil engraftment [124].

Another small study reported that haploidentical MSCs infused in conjunction with allogeneic hematopoietic stem cell transplantation led to enhanced engraftment. In addition, all patients achieved neutrophil and platelet engraftment and 100% donor chimerism, again with the noted absence of any associated toxicity [125]. Lazarus et al. [126] reported the use of MSCs in an open-label, multicenter trial in patients with hematologic malignancy. This involved the coadministration of culture-expanded MSCs with HLA-identical sibling-matched HSCs. The authors reported that culture-expanded MSCs together with HSC transplantation was a safe procedure and could potentially reduce transplant side effects and enhance marrow recovery after myeloablative treatment.

In another small pilot study, Gonzalo-Daganzo et al. [127] reported that MSCs were better employed prophylactically when used to treat acute GvHD in umbilical cord blood transplantation. Chen et al. [118] reported that intracoronary injection of MSCs in patients with AMI resulted in significantly increased wall movement velocity over the infarcted area in MSC-treated patients. Most notably, left ventricular ejection fraction was also higher in the MSC-treated group compared with controls at 3 months follow-up observations.

In addition, significant efforts have been expended in MSC therapies from industry experts. Osiris Therapeutics Inc. (Columbia, MD, USA, http://www.osiristx.com/clinical_trials.php) is currently evaluating their proprietary adult stem cell product, Prochymal, in phase III clinical trials for three indications, including steroid refractory acute graft-versus-host disease (GvHD), newly diagnosed acute GvHD, and Crohn's disease. Prochymal are adult human MSCs derived from healthy donors. Osiris is also evaluating Prochymal in phase II clinical trials for type 1 diabetes mellitus and chronic obstructive pulmonary disease. Follow-up data from the phase II trials for the treatment of acute GvHD showed that 74% of patients experienced total clinical resolution of the disease, whereas follow-up data for the phase II trials for the treatment of Crohn's disease for patients who had failed to respond to standard treatments, such as steroids, reported a significant reduction in disease severity by day 28 with relatively low doses of Prochymal and a short treatment course. Athersys (Cleveland, OH, USA, http://www.athersys.com) is another industry-led company investigating the therapeutic potential of MSCs. Athersys is currently evaluating the potential of MultiStem (progenitor cells harvested from a prequalified donor) in several phase I clinical trials for ischemic injury (myocardial infarction, stroke, and other indications) and conditions involving the immune system (autoimmune disease).

At the time of writing this review, according to the clinical trials Website of the United States sponsored by the National Institutes of Health (http://clinicaltrials.gov), approximately 80 clinical trials are currently exploring the application of MSCs. In addition to the application of MSCs for renal and CVD pathologies, the use of MSCs is also actively pursued in a diverse range of other conditions, including hematologic pathologies such as graft-versus-host disease (GvHD), osteogenesis imperfecta, amyotrophic lateral sclerosis (ALS), Hurler syndrome, metachromatic leukodystrophy, and Crohn's disease. In addition, MSC transplants have been investigated to improve recovery after myeloablative therapy for treatment of solid tumors. Table 2 summarizes some of the current clinical trials using MSCs.

### Future Directions

The last few years have witnessed a growing optimism by both basic scientists and clinicians for the clinical application of MSCs for many disease pathologies. Tremendous advancements have been made from significant in vitro and in vivo preclinical studies using MSCs. Although MSCs were originally heralded for their ability to contribute to tissue regeneration through engraftment and long-term survival in injured tissues via their presumed plasticity, recent findings have suggested a plethora of additional mechanisms through which MSCs exert their seemingly beneficial effects, including immunomodulation and paracrine processes. In addition, the increasing number of clinical trials demonstrating the absence of any major adverse side effects coupled with early optimistic benefits continues to drive the field of MSC therapy. However, unresolved issues such as the lack of conformity with respect to isolation and ex vivo culture-expansion protocols and the heterogeneity by which populations and subpopulations of MSCs are characterized continue to be obstacles. In addition, the conflicting data regarding the ability of MSCs to engraft and differentiate into functional cardiomyocytes or tubular epithelial cells, as well as numerous studies reporting the beneficial effects of MSCs in early time frames, suggest that the benefits are solely attributable to paracrine mediated effects. It is clear that much more work is needed and evidence from long-term studies is absolutely required to validate the nature of MSC-based therapy before the prospect of developing a genuine candidate for an “off-the-shelf” MSC biotherapeutic product is achievable.
